# Beyond cytotoxicity: molecular mechanisms of natural products as multi-targeting modulators and chemo-sensitizers in oncology

**DOI:** 10.3389/fphar.2026.1782874

**Published:** 2026-03-27

**Authors:** Jinshuo Ma, Xue Wang, Qiang Sun

**Affiliations:** 1 School of Pharmacy, North Henan Medical University, Xinxiang, Henan, China; 2 School of Life Sciences and Technologies, North Henan Medical University, Xinxiang, Henan, China

**Keywords:** cancer therapy, chemosensitization, drug resistance, multi-targeting agents, natural products, synergistic therapy, tumor microenvironment

## Abstract

Natural products have historically anchored anticancer drug discovery, providing scaffold structures for essential agents like taxanes and vinca alkaloids. While these compounds are traditionally valued for their direct cytotoxicity, this reductionist perspective overlooks their capacity to modulate the intricate, redundant signaling networks that drive malignancy. Conventional chemotherapy is increasingly constrained by dose-limiting toxicities and the emergence of multidrug resistance (MDR), often driven by transporter-mediated efflux and apoptosis evasion. This mini-review advances a multi-targeting perspective, re-positioning natural products not merely as cell-killing agents, but as broad-spectrum agents and synergistic chemo-sensitizers. We critically examine evidence demonstrating how specific flavonoids, terpenoids, and alkaloids simultaneously disrupt multiple cancer hallmarks—including aberrant PI3K/Akt/mTOR signaling, cell cycle progression, and the pro-tumorigenic microenvironment—thereby preventing compensatory pathway activation. Furthermore, we elucidate their translational utility as chemo-sensitizers. Mechanistically, these agents can inhibit ATP-binding cassette (ABC) transporters, lower the apoptotic threshold by neutralizing survivin, and mitigate chemotherapy-induced organ damage. By integrating natural products into rational, evidence-based combination regimens, it may be possible to enhance the therapeutic index of standard-of-care drugs in specific preclinical models. This review argues that overcoming pharmacokinetic barriers and standardization issues will allow these pleiotropic agents to transition from complementary additives to integral components of precision oncology.

## 1 Introduction

Cancer remains a leading cause of global mortality, necessitating a continuous evolution of therapeutic strategies (Siegel et al., 2023; Sung et al., 2021). While the pillars of modern oncology—chemotherapy, radiotherapy, targeted therapy, and immunotherapy—have improved survival rates, significant limitations persist (Hanahan, 2022; Vanneman and Dranoff, 2012). Chemotherapy, a mainstay for decades, is restricted by a narrow therapeutic index (Chabner and Roberts, 2005; DeVita et al., 2008). Cytotoxic agents targeting rapidly dividing cells inevitably damage healthy, high-turnover tissues, resulting in dose-limiting toxicities such as myelosuppression and mucositis (Nurgali et al., 2018; Schirrmacher, 2019).

A critical impediment to durable clinical success is multidrug resistance (MDR) (Gottesman et al., 2002; Vasan et al., 2019). Whether intrinsic or acquired, MDR renders tumors cross-resistant to structurally unrelated agents, leading to treatment failure (Holohan et al., 2013). The mechanisms driving MDR are multifactorial, involving the overexpression of ATP-binding cassette (ABC) transporters (e.g., P-glycoprotein), (Szakacs et al., 2006; Robey et al., 2018), enhanced DNA repair, metabolic detoxification, and the deregulation of apoptotic pathways (Bukowski et al., 2020; Carneiro and El-Deiry, 2020).

Historically, natural products provided the scaffold for essential drugs like paclitaxel and camptothecin, selected primarily for their potent cytotoxicity (Koehn and Carter, 2005; Newman and Cragg, 2020). However, a nuanced pharmacological perspective is emerging. Rather than serving solely as cell killers, many natural compounds function as biological response modifiers (Efferth and Koch, 2011; Atanasov et al., 2021). At sub-toxic concentrations, these agents exert pleiotropic effects, modulating interconnected signaling nodes within the tumor network (Aggarwal et al., 2006; Hopkins, 2008). This contrasts with the magic bullet approach of specific targeted therapies, which are often circumvented by compensatory bypass signaling (Strebhardt and Ullrich, 2008; Gertsch, 2011).

This mini-review explores the evolving role of natural products in oncology. We move beyond the classical cytotoxic paradigm to examine their capacity as multi-targeting modulators of cancer hallmarks and their translational potential as synergistic chemo-sensitizers. This multi-targeted approach offers a rational basis for developing more effective and tolerable combination therapies.

## 2 Natural products as multi-targeting modulators of cancer signaling networks

**FIGURE 1:**
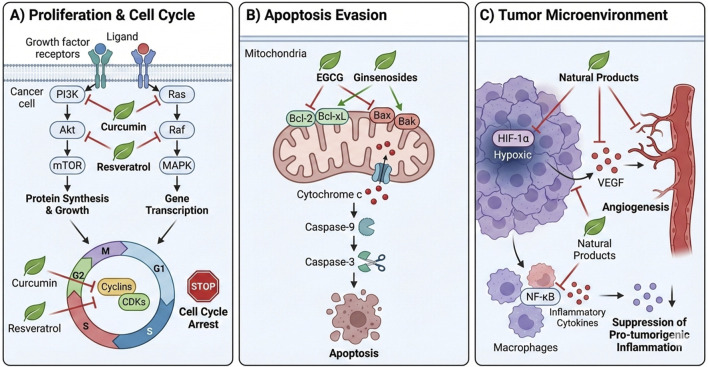
The Multi-Targeting Strategy of Natural Products Against the Hallmarks of Cancer. This schematic illustrates the pleiotropic effects of natural products across three core domains: **(A)** Proliferation and Cell Cycle Control: Detailed inhibition of the PI3K/Akt/mTOR and MAPK cascades by curcumin and resveratrol, leading to G1/S arrest. **(B)** Apoptosis Restoration: The shift from anti-apoptotic (Bcl-2/Mcl-1) to pro-apoptotic (Bax/Bak) dominance induced by ginsenosides and EGCG, triggering the caspase cascade. **(C)** Tumor Microenvironment Modulation: Suppression of angiogenesis via the HIF-1α/VEGF axis and attenuation of pro-tumorigenic inflammation through NF-κB inhibition.

The resilience of malignant cells arises from robust, redundant signaling networks (Kitano, 2004). Unlike targeted therapies that inhibit single nodes, natural products often act as multi-targeting agents, exerting moderate but simultaneous effects on multiple targets (Csermely et al., 2005). This broad-spectrum modulation complicates the cancer cell’s ability to develop resistance via single-pathway mutations (Keith et al., 2005) (Figure 1).

### 2.1 Modulating proliferative signaling and the cell cycle machinery

Sustained proliferative signaling is a foundational cancer hallmark (Hanahan and Weinberg, 2011), driven by autocrine growth factor production, receptor overexpression, or the constitutive activation of intracellular conduits such as the PI3K/Akt/mTOR and Ras/Raf/MAPK pathways (Manning and Cantley, 2007; Roberts and Der, 2007). Natural products can effectively intercept these signals at multiple regulatory nodes (Gupta et al., 2010).

Curcumin exemplifies multi-level pathway inhibition. It significantly inhibits the PI3K/Akt/mTOR axis by inhibiting the catalytic activity of PI3K and PDK1 (Johnson and Mukhtar, 2007). This prevents Akt phosphorylation at critical regulatory sites (Thr308 and Ser473), effectively severing signaling to mTORC1. Consequently, downstream effectors—p70S6K and the translational repressor 4E-BP1—are dysregulated, halting the protein synthesis necessary for division (Shehz et al., 2013). Simultaneously, curcumin disrupts the Ras/Raf/MEK/ERK cascade and inhibits oncogenic transcription factors often constitutively active in malignancy, including STAT3 (blocking phosphorylation and dimerization) (Bharti et al., 2003) and NF-κB (via IKK suppression) (Si et al., 1995). These convergent actions suppress the expression of Cyclin D1, a master regulator of the G1-S transition (Kunnumakkara et al., 2017).

Resveratrol exerts antiproliferative effects linked to cell cycle arrest. By inhibiting Cyclin D/CDK4 and Cyclin E/CDK2 complexes, it reinforces the G1 checkpoint (Ahmad et al., 2001). Mechanistically, this is achieved by upregulating the CDK inhibitors p21WAF1 and p27KIP1, which maintains the Retinoblastoma protein (Rb) in an active, hypophosphorylated state (Ahmad et al., 2001). Hypophosphorylated Rb sequesters the E2F transcription factor family, preventing the activation of S-phase genes such as DNA polymerases and thymidine kinase (Ahmad et al., 2001). Furthermore, resveratrol activates the metabolic sensors AMPK and SIRT1 (Howitz et al., 2003); this axis indirectly suppresses proliferation by inhibiting mTOR signaling and deacetylating key regulatory factors like p53 and FOXO proteins (Canto and Auwerx, 2009; Yi and Luo, 2010).

Other agents provide complementary mechanisms. Berberine acts as a metabolic brake, activating AMPK to inhibit mTORC1-driven growth (Lee et al., 2006; Liu et al., 2015). Genistein functions as a broad-spectrum tyrosine kinase inhibitor, competitively targeting the ATP-binding pockets of receptor tyrosine kinases (e.g., EGFR) and non-receptor kinases (e.g., Src), thereby blocking signal transduction at its inception (Akiyama et al., 1987; Spagnuolo et al., 2015).

### 2.2 Reshaping the balance of apoptosis and autophagy

Natural products can skillfully restore apoptotic sensitivity by re-calibrating the cell’s life-death rheostat. The intrinsic, or mitochondrial, pathway of apoptosis is governed by the intricate interplay of the Bcl-2 protein family (Czabotar et al., 2014). This family consists of anti-apoptotic members (e.g., Bcl-2, Bcl-xL, Mcl-1), which preserve mitochondrial integrity, and pro-apoptotic members, which are further divided into the effectors (Bax, Bak) that directly permeabilize the mitochondrial outer membrane, and the BH3-only proteins (e.g., Bid, Bim, Puma) that act as sensors of cellular stress and activators of the effectors. The ratio of these proteins determines a cell’s fate (Oltvai et al., 1993). Ginsenosides, from *Panax ginseng*, are effective modulators of this ratio. They can transcriptionally repress Bcl-2 and Mcl-1 while simultaneously upregulating Bax and BH3-only proteins like Puma and Noxa (Wang et al., 2007; Nag et al., 2012). This decisive shift in the balance triggers Bax/Bak oligomerization, leading to mitochondrial outer membrane permeabilization (MOMP), the release of cytochrome c and Smac/DIABLO into the cytoplasm, and the subsequent activation of the initiator caspase-9 and executioner caspase-3 (Wong et al., 2015).

Furthermore, many natural compounds, including betulinic acid and various terpenoids, can directly induce the production of reactive oxygen species (ROS) within mitochondria. This oxidative stress can cause direct damage to mitochondrial DNA and lipids, serving as a powerful, independent trigger for MOMP (Fulda et al., 1998). The extrinsic pathway, initiated by the binding of ligands like FasL or TRAIL to their cognate death receptors, can also be modulated. Ursolic acid, a triterpenoid found in apples and rosemary, has been shown to upregulate the expression of death receptors DR4 and DR5 on the surface of cancer cells, making them highly sensitive to TRAIL-mediated apoptosis (Chen C. J. et al., 2019). Adding another layer of complexity, compounds like Shikonin, from the root of *Lithospermum erythrorhizon*, can induce necroptosis, a regulated, inflammatory form of necrosis, by targeting pyruvate kinase M2 (PKM2), thus offering an alternative cell death route when apoptosis is blocked (Han et al., 2007; Chen et al., 2011).

Autophagy’s role in cancer is a classic example of biological context-dependency (Levine and Kroemer, 2008; White, 2012). While it can suppress tumor initiation, in established tumors it frequently functions as a cytoprotective mechanism, allowing cancer cells to survive nutrient deprivation and chemotherapy-induced stress (Rubinsztein et al., 2007). The strategic modulation of autophagy is therefore a promising therapeutic approach. When chemotherapy induces a protective autophagic response, co-treatment with natural products can be highly effective. Rottlerin, a polyphenol from *Mallotus philippensis*, and 3-Methyladenine (3-MA), although a synthetic tool compound, serve as conceptual models for inhibiting the initial stages of autophagy by targeting the class III PI3K, Vps34 (Amaravadi et al., 2011). Curcumin and resveratrol have also been shown to block the autophagic flux in certain contexts, preventing the degradation of cellular components and leading to the accumulation of dysfunctional autophagosomes, which ultimately sensitizes cells to apoptosis (Bousset and Gil, 2022). Conversely, compounds like artemisinin and its derivatives can induce massive, lethal autophagy, representing a therapeutic strategy for cancers that are inherently resistant to apoptosis (Hamacher-Brady et al., 2011).

### 2.3 Inhibiting the tumor microenvironment: angiogenesis, inflammation, and metastasis

A tumor cannot thrive in isolation. It actively co-opts and corrupts its surrounding environment—the TME—to support its growth, vascularization, and dissemination. Natural products are particularly adept at targeting this complex ecosystem (Hanahan, 2022; Hanahan and Weinberg, 2011).

Anti-angiogenesis: As tumors expand, they require a dedicated blood supply, a process driven by angiogenesis (Folkman, 1971). The master regulator of this process under hypoxic conditions is HIF-1α. (Semenza, 2003) In normoxic cells, HIF-1α is hydroxylated and targeted for proteasomal degradation by the VHL E3 ubiquitin ligase. Under hypoxia, this degradation is blocked, allowing HIF-1α to accumulate, translocate to the nucleus, and activate the transcription of pro-angiogenic genes, most notably VEGF (Jaakkola et al., 2001; Kaelin, 2002). Natural products like genistein and luteolin inhibit this axis at multiple levels. They can promote the VHL-independent degradation of HIF-1α, inhibit its transcriptional activity, and also suppress the downstream signaling of the VEGF receptor (VEGFR2) in endothelial cells, thereby preventing their proliferation, migration, and tube formation (Bagli et al., 2004; Xie et al., 2014). Andrographolide, from *Andrographis paniculata*, is another potent inhibitor of angiogenesis, acting primarily by suppressing NF-κB-mediated VEGF production (Chen et al., 2004; Peng et al., 2018).

Anti-inflammation: The link between chronic inflammation and cancer is now irrefutable. The inflammatory TME is populated by immune cells that, instead of attacking the tumor, are often reprogrammed to secrete a cocktail of growth factors, cytokines (e.g., TNF-α, IL-6), and enzymes that promote tumor progression (Grivennikov et al., 2010). As discussed, NF-κB is a central node in this process (Karin, 2006). Beyond curcumin and parthenolide, Boswellic acids, from the resin of *Boswellia* species (frankincense), inhibit a different branch of the inflammatory cascade. They are direct, non-redox inhibitors of 5-lipoxygenase (5-LOX) (Safa et al., 1992), a key enzyme in the synthesis of pro-inflammatory leukotrienes, which have been implicated in the pathogenesis of several cancers (Ammon, 2016).

Anti-metastasis: The metastatic cascade is a multi-step process that involves local invasion, intravasation, survival in circulation, extravasation, and colonization of a distant site. A key initiating event is the epithelial-mesenchymal transition (EMT), a process where epithelial cancer cells lose their polarity and cell-cell adhesion and gain migratory and invasive properties. This is orchestrated by transcription factors like Snail, Slug, and ZEB1 (Thiery, 2002; Kalluri and Weinberg, 2009). Curcumin and EGCG have been shown to reverse EMT by upregulating the epithelial marker E-cadherin while downregulating mesenchymal markers like N-cadherin and Vimentin, effectively gluing the cancer cells back in place (Bao et al., 2012; Oh et al., 2014). To invade surrounding tissues, cancer cells must degrade the extracellular matrix (ECM) by secreting matrix metalloproteinases (MMPs), such as MMP-2 and MMP-9. A vast number of natural products, including flavonoids, catechins, and triterpenoids, have been shown to inhibit the expression and activity of these MMPs, thus blocking a critical step in both local invasion and metastasis (Demeule et al., 2000; Roomi et al., 2006).

### 2.4 Rewiring cancer metabolism and redox homeostasis

Emerging evidence indicates that natural products are potent regulators of cancer metabolic rewiring, effectively disrupting the altered energy dynamics that sustain rapid proliferation. Beyond kinase inhibition, many compounds directly target the Warburg effect and mitochondrial bioenergetics. A comprehensive example is the action of cannabinoids, which have been recently demonstrated to profoundly modulate glycolysis, mitochondrial oxidative phosphorylation, lipid metabolism, and redox homeostasis in various cancer model (Sun et al., 2023). By shifting the metabolic phenotype and disrupting redox balance, these natural bioactive compounds deprive cancer cells of essential biosynthetic precursors and ATP, creating a metabolic vulnerability that enhances the efficacy of subsequent therapeutic interventions.

## 3 Natural products as synergistic chemo-sensitizers

The most immediate clinical application of natural products lies in their role as chemo-sensitizers. Rather than standalone cures, they function to enhance the efficacy of conventional chemotherapy and mitigate toxicity, addressing the dual challenges of resistance and safety.

### 3.1 Reversing multidrug resistance

The overexpression of ABC transporters is a primary driver of MDR, acting as molecular pumps that actively expel chemotherapeutic drugs from the cancer cell. The development of synthetic ABC transporter inhibitors has been largely disappointing, with multiple generations of drugs failing in clinical trials due to unacceptable toxicity or lack of efficacy (Robey et al., 2018). This has renewed interest in natural products as a source of safer and more effective MDR modulators (Kibria et al., 2014).

P-glycoprotein (P-gp/ABCB1), the first discovered and best-characterized transporter, is a hydrophobic vacuum cleaner with broad substrate specificity, capable of effluxing drugs like doxorubicin, paclitaxel, and vincristine (Aller et al., 2009). As noted, quercetin functions as a competitive inhibitor. Its planar structure allows it to dock within the large drug-binding pocket of P-gp, physically obstructing the binding of chemotherapeutic agents (Chen et al., 2010). It can also interfere with the transporter’s ATP hydrolysis cycle, starving it of the energy required for transport. Beyond direct inhibition, quercetin can also modulate the lipid environment of the cell membrane, which can allosterically affect P-gp’s conformational state and function. Silymarin and its active component, silibinin, provide a more comprehensive approach. In addition to functional inhibition, they transcriptionally repress the *ABCB1* gene by suppressing the PI3K/Akt/NF-κB signaling pathway, which is known to be a positive regulator of *ABCB1* expression (Molavi et al., 2017). This reduction in the total amount of P-gp protein provides a more sustained reversal of resistance.

The repertoire of natural MDR modulators is vast. Tetrandrine, a bisbenzylisoquinoline alkaloid from *Stephania tetrandra*, is one of the most potent non-competitive P-gp inhibitors known (Fu et al., 2004). Ginsenoside Rg3, a specific saponin from processed ginseng, has been shown to effectively reverse P-gp-mediated resistance to both doxorubicin and paclitaxel in various cancer models (Kim et al., 2003). Furthermore, resistance is not solely a P-gp problem. Other transporters like MRP1 (ABCC1), which effluxes conjugated drugs, and BCRP (ABCG2), which effluxes topotecan and mitoxantrone, also play critical roles. Excitingly, many natural products exhibit broad-spectrum inhibitory activity. Curcumin, for instance, has been shown to inhibit not only P-gp but also MRP1 and BCRP, making it a particularly attractive candidate for combating complex MDR phenotypes (Sharma et al., 2009).

### 3.2 Sensitizing apoptosis-resistant cells

The ultimate goal of most chemotherapy is to induce apoptosis. Cancer cells counter this by erecting a powerful anti-apoptotic blockade, primarily through the overexpression of Bcl-2 family proteins and IAP family proteins (Pistritto et al., 2016). Natural products can dismantle this blockade, effectively lowering the apoptotic threshold and “priming” the cells for chemotherapy-induced death.

The role of berberine in downregulating survivin provides a clear example. Survivin is a unique IAP that not only inhibits caspases but also regulates microtubule dynamics during mitosis. Its overexpression is a strong predictor of poor outcomes and resistance to taxanes (Altieri, 2003). By suppressing survivin transcription, berberine removes a critical brake on both the apoptotic machinery and cell cycle progression. When combined with a drug like paclitaxel, this leads to synergistic effects: paclitaxel induces mitotic arrest, a state where survivin levels are normally high to prevent cell death, but berberine’s action ensures that this arrest is lethal, culminating in robust apoptosis (Wu et al., 1999; Zhao et al., 2016).

Honokiol, the lignan from *Magnolia* bark, directly targets the anti-apoptotic Bcl-2 proteins. It is known to bind to a hydrophobic groove on the surface of Bcl-xL and Mcl-1, preventing them from sequestering pro-apoptotic proteins like Bax and Bak (Pan et al., 2016). This action is functionally analogous to that of synthetic BH3-mimetic drugs like venetoclax. By pre-treating cells with honokiol, one effectively “unleashes” Bax and Bak. Subsequent damage from a chemotherapeutic agent then easily triggers full-blown MOMP and apoptosis. Another powerful agent is Gambogic Acid, a xanthonoid from *Garcinia hanburyi* resin. It covalently binds to a cysteine residue in the BH3-binding pocket of Bcl-2, irreversibly inactivating it (Zhai et al., 2008). It also potently inhibits the proteasome, causing the accumulation of pro-apoptotic proteins and further sensitizing the cell (Bazzaro et al., 2008).

Withaferin A, a steroidal lactone from Ashwagandha (*Withania somnifera*), employs a different strategy. It inhibits the chaperone protein Hsp90 (Yu et al., 2010). Hsp90 is required for the proper folding and stability of numerous oncoproteins, including Akt, HER2, and mutant p53. By inhibiting Hsp90, Withaferin A triggers the proteasomal degradation of these key survival proteins, effectively dismantling the entire pro-survival signaling network and leaving the cancer cell highly vulnerable to conventional cytotoxic agents (Xing et al., 2023).

### 3.3 Alleviating chemotherapy-induced toxicity

Improving the therapeutic index of chemotherapy by selectively protecting normal tissues from damage is a critical goal of integrative oncology. The potent antioxidant and anti-inflammatory properties of natural products make them ideal candidates for this role.

Cardiotoxicity and Nephrotoxicity: The mechanism of doxorubicin cardiotoxicity is complex, involving ROS generation via redox cycling of the drug’s quinone moiety and its interaction with iron, as well as the induction of DNA double-strand breaks in cardiomyocytes through its interaction with the Topoisomerase IIβ isoform (Zhang et al., 2012). Resveratrol offers multi-level protection. Its powerful antioxidant activity quenches ROS, while its activation of the Nrf2 pathway boosts the cell’s endogenous antioxidant defenses (e.g., superoxide dismutase, catalase). Its activation of SIRT1 improves mitochondrial function and reduces cell death (Chen et al., 2011; Chen et al., 2024). Importantly, this protection appears to be selective for cardiomyocytes, without compromising doxorubicin’s anti-tumor effect, which is primarily mediated via Topoisomerase IIα. Similarly, cisplatin nephrotoxicity is driven by intense oxidative stress and inflammation in renal proximal tubule cells. This involves the activation of pathways like TLR4 and TNF-α (Pabla and Dong, 2008). Natural polyphenols like EGCG and curcumin can effectively interrupt this toxic cascade by scavenging ROS, chelating free metal ions, and potently inhibiting the NF-κB and MAPK pathways that drive inflammation in renal tissue (Kuhad et al., 2007; Chen et al., 2015).

Neurotoxicity and CINV: Platinum- and taxane-induced peripheral neuropathy is a debilitating, often irreversible side effect. The underlying mechanism involves damage to mitochondria and induction of oxidative stress in dorsal root ganglia neurons (Areti et al., 2014). Preclinical studies suggest that compounds capable of improving mitochondrial health and reducing oxidative stress, such as Ginkgo Biloba Extract and acetyl-L-carnitine, may be protective. As mentioned, gingerol is a clinically validated antiemetic for CINV. Its mechanism is multifaceted, including 5-HT3 receptor antagonism and anti-inflammatory effects in the gut (Walstab et al., 2013; Marx et al., 2017). Other compounds like capsaicin, from chili peppers, can desensitize afferent C-fibers by acting on the TRPV1 receptor, potentially modulating the transmission of nausea signals and substance P release. The use of these agents as supportive care can dramatically improve a patient’s ability to tolerate and complete their prescribed chemotherapy regimen (Table 1).

**TABLE 1:** Synergistic combinations of natural products with conventional chemotherapeutic agents.

Natural products (source)	Conventional drug	Cancer type(s)	Principal synergistic mechanism of action	Evidence level	Key references
Quercetin (Fruits, Vegetables)	Doxorubicin, Paclitaxel	Breast, Ovarian	Overcoming MDR: Competitive inhibition of P-glycoprotein (P-gp) function, increasing intracellular drug accumulation	*in vitro/in vivo*	Chen et al. (2010), Li et al. (2018)
Curcumin (*Curcuma longa*)	Cisplatin, Gemcitabine	Pancreatic, Lung	Enhancing Apoptosis and Overcoming MDR: Downregulation of NF-κB and STAT3 survival signals; Inhibition of P-gp, MRP1, and BCRP.	*in vitro/in vivo*	Kunnumakkara et al. (2017), Anitha et al. (2014)
EGCG (Green Tea)	Doxorubicin, Cisplatin	Various (Cardio/Nephro)	Mitigating Toxicity: Potent antioxidant activity; Iron chelation (for doxorubicin); Inhibition of inflammatory pathways in normal tissues	*in vivo*	Sahin et al. (2010), Singh et al. (2011)
Silymarin (*Silybum marianum*)	Doxorubicin, Cisplatin	Breast, Prostate	Overcoming MDR: Functional inhibition and transcriptional downregulation of P-gp via Akt/NF-κB pathway suppression	*in vitro*	Zhang et al. (2018), Delmas et al. (2020)
Resveratrol (Grapes, Berries)	Doxorubicin	Cardiomyocytes	Mitigating Toxicity: Activation of SIRT1 and Nrf2 pathways, enhancing endogenous antioxidant defenses and mitochondrial function in the heart	*in vivo*	Zhang et al. (2011), Sin et al. (2015)
Berberine (*Coptis chinensis*)	Paclitaxel, Cisplatin	Ovarian, Breast	Enhancing Apoptosis: Transcriptional suppression of the anti-apoptotic protein survivin, lowering the threshold for drug-induced cell death	*in vitro*	Wu et al. (1999), Zhao et al. (2016)
Honokiol (*Magnolia officinalis*)	Gemcitabine, Cisplatin	Pancreatic, Lung	Enhancing Apoptosis: Direct inhibition of anti-apoptotic proteins Bcl-xL and Mcl-1, functioning as a natural BH3-mimetic	*in vitro/in vivo*	Pan et al. (2016), Arora et al. (2012)
Gingerol (*Zingiber officinale*)	Various	Clinical (Supportive Care)	Mitigating Toxicity: Antagonism of 5-HT3 receptors in the gut and CNS, providing clinically validated anti-emetic effects (CINV)	Clinical Trial	Marx et al. (2017), Ryan et al. (2012)

Abbreviations: MDR, multidrug resistance; P-gp, P-glycoprotein; NF-κB, Nuclear Factor kappa B; STAT3, Signal Transducer and Activator of Transcription 3; MRP1, Multidrug Resistance-associated Protein 1; BCRP, breast cancer resistance protein; SIRT1, Sirtuin 1; Nrf2, Nuclear factor erythroid 2-related factor 2; Bcl-xL, B-cell lymphoma-extra large; Mcl-1, Myeloid cell leukemia 1; BH3, Bcl-2, homology domain 3; CNS, central nervous system; CINV, Chemotherapy-Induced Nausea and Vomiting; 5-HT3, 5-Hydroxytryptamine 3 (Serotonin).

### 3.4 Translational readiness and real-world clinical concerns

While preclinical data strongly advocate for natural products as chemo-sensitizers, a critical distinction must be made between *in vitro* phenomena, *in vivo* efficacy, and clinical outcomes. Many compounds exhibit striking synergistic cytotoxicity in cell culture but fail in animal models or clinical trials due to poor bioavailability, rapid clearance, or off-target effects.

Furthermore, real-world clinical integration must address substantial safety concerns and complex drug-herb interactions. First, many natural products (e.g., St. John’s wort, curcumin) strongly modulate Cytochrome P450 (CYP) enzymes and drug transporters, which can unpredictably alter the pharmacokinetics of co-administered chemotherapeutics, risking either systemic toxicity or sub-therapeutic plasma levels (Duan et al., 2025). Second, clinical oncologists frequently raise concerns regarding heightened risks of platelet dysfunction, bleeding events, and QT interval prolongation associated with high-dose botanical extracts. Finally, a long-debated pharmacokinetic antagonism exists regarding antioxidants: while natural antioxidants may mitigate collateral tissue damage, they may theoretically compromise the efficacy of ROS-mediated treatments—such as anthracyclines, platinum compounds, and radiotherapy—by scavenging the critical free radicals required to induce DNA damage and tumor cell death in specific contexts (Woldeselassie and Tamene, 2024). Therefore, transitioning from *in vitro* synergy to translational readiness strictly requires rigorous pharmacokinetic profiling and context-dependent clinical validation.

## 4 Discussion

The evidence presented supports a pharmacological re-evaluation of natural products in oncology. Their value lies not primarily as potent cytotoxins, but as multi-targeting modulators that alter biological networks to destabilize cancer phenotypes and amplify the efficacy of conventional drugs. This dual functionality provides a compelling rationale for their integration into modern regimens.

However, clinical translation faces substantial hurdles. The whole extract versus single compound debate persists. While extracts offer phytochemical synergy (entourage effect), they present challenges in standardization and regulatory approval (Rasoanaivo et al., 2011; Caesar and Cech, 2019). Purified compounds offer precision but often suffer from poor pharmacokinetics. Curcumin illustrates the bioavailability paradox: despite high *in vitro* potency, its poor solubility and rapid metabolism result in negligible systemic exposure, limiting clinical efficacy (Anand et al., 2007; Nelson et al., 2017).

Future research must prioritize two areas to bridge the translational gap. First, advanced drug delivery systems are non-negotiable to overcome the inherent pharmacokinetic barriers of natural products (Pistritto et al., 2016; Bonifacio et al., 2014). Nanotechnology can resolve solubility issues and protect compounds from rapid degradation. More importantly, next-generation delivery systems are actively exploiting the unique pathophysiological features of the TME—such as hypoxia, elevated reactive oxygen species, and mild acidity—to achieve on-demand drug release. For example, recent developments in TME-responsive nanoplatforms have enabled highly precise tumor-cell targeting and multimodal therapy combinations (Wang et al., 2024). A concrete mechanistic illustration is the development of spermine-modified polymeric micelles; these smart nanocarriers remain stable in systemic circulation but rapidly undergo charge-reversal and disassemble upon encountering the acidic extracellular pH of the tumor microenvironment (pH ∼6.5), thereby triggering spatially controlled drug release and significantly enhancing deep tumor penetration (Chen Y. et al., 2019). Second, while this review emphasizes molecular mechanisms, identifying the optimal multi-target synergies out of thousands of natural compounds cannot rely solely on empirical screening. Moving forward, network pharmacology and computational systems biology must be integrated as concrete methodological tools to map target spaces and predict compound-drug interactions. The most robust future studies will adopt a dual-pronged approach, actively combining *in silico* network predictions with rigorous experimental validation, as recently demonstrated by successful models identifying network-based therapeutic targets for naringenin in cervical cancer (Kibble et al., 2015; Huang et al., 2026; Zhou et al., 2024). By merging predictive algorithms with precise TME-responsive delivery, natural products can transition from complementary add-ons to highly targeted components of precision oncology.

Furthermore, research must expand to emerging frontiers, such as targeting cancer stem cells (CSCs) and modulating the gut microbiome, which influences the metabolism and activity of systemic drugs (Feng et al., 2019). Finally, the potential of natural products to remodel the immune microenvironment—turning “cold” tumors “hot”—suggests a promising synergy with immune checkpoint inhibitors (Galon and Bruni, 2019; Dong et al., 2022).

In conclusion, by applying rigorous pharmacological standards and leveraging technological advancements to overcome pharmacokinetic limitations, natural products can transition from complementary add-ons to integral components of evidence-based integrative oncology. This evolution promises therapeutic strategies that are not only more effective but significantly more tolerable for patients.
